# Editorial Note

**Published:** 1927

**Authors:** 


### Editorial Note.

**Richard Bright, 1789–1858.**

On July 8th a famous Bristolian was commemorated at Guy's Hospital and at the Royal College of Physicians. Richard Bright, whose name is perpetuated in "Bright's Disease," was born in Bristol in 1789. His father, Richard Bright, was an influential banker and prominent citizen of Bristol, who resided at Ham Green House. His third son, Richard, was educated at Dr. Estlin's School, and subsequently at Edinburgh University and Guy's Hospital. He took the degree of M.D. at Edinburgh in 1813, and in 1820 was appointed Assistant Physician at Guy's, becoming in due course Physician. But ill-health caused his retirement from the Hospital in 1843 at the early age of 54. His remaining years were devoted to consulting practice, to travel and to his many artistic interests. In spite of ill-health his practice, which had been slow in coming, grew to be perhaps the largest in London. He died after a short final illness on December 14th, 1858.

The occasion of the commemorative celebrations in London was the centenary of the publication of the first volume of Bright's *Reports of Medical Cases Selected with a View of Illustrating the Symptoms and Cure of Diseases by a Reference to Morbid Anatomy.* It contains the announcement of the discovery of

"Bright's Disease." The completeness of Bright's account of those forms of renal disease associated with his name will be realised from the fact that he described the uræmic symptoms, cerebral hemorrhage, loss of sight, the hard pulse, œdema, pleural, pericardial and peritoneal effusions, bronchitis, cardiac hypertrophy and the enteritis which may attend the disease. But even if he had not discovered the disease which bears his name he would have been, as the *British Medical Journal* observes, one of the greatest of our physicians. His powers of observation were marvellous —none of his observations have been shown to be incorrect. He could write well; as Sir William Hale-White said in the account of Bright's life he contributed to the *Guy's Hospital Reports* in 1921: "Many modern text-books would be vastly improved if some of their pages were deleted to make room for Bright's descriptions." He was an artist too—no medical work has more beautiful pictures than are to be found in the *Reports of Medical Cases.*

In the words of Professor Thayer's address at Guy's Hospital on July 8th: "Happy in his birth and in the surroundings and conditions of his early life, cultivated by travel and society, an artist, a linguist, a man of scholarly tastes, Bright made full use of his opportunities." Bristol may claim some small share in furnishing at least the "opportunities" of his birth and surroundings of his early life, and our city hospital at Ham Green may gather some reflected lustre from having been the home of his childhood.

